# Supplementary material for: Disengaging spinal afferent nerve communication with the brain in live mice
Source: Commun Biol. 2022 Sep 14;5:915. doi: 10.1038/s42003-022-03876-x (PMC9475039; doi:10.1038/s42003-022-03876-x)
Supplement: Supplementary file 2 — Supplementary information [file 42003_2022_3876_MOESM2_ESM.pdf]

	Control		LS lesion		P (time x treatment)	F	DFn, DFd
	Baseline	Post surg.	Baseline	Post Surg.			
Bodyweight (g)	22.9 ± 1.6	22.9 ± 1.6	22.7 ± 1.3	22.7 ± 1.3	0.923	0.0078	1, 8
Food intake(g)	3.7 ± 0.2	3.8 ± 0.3	3.7 ± 0.3	3.6 ± 0.3	0.514	0.4654	1, 8
Water intake (ml)	4.4 ± 0.7	5.4 ± 0.8	4.4 ± 0.7	5.1 ± 0.7	0.400	0.7913	1, 8
Faeces (dry g)	0.6 ± 0.03	0.8 ± 0.04	0.7 ± 0.10	0.8 ± 0.08	0.432	0.6844	1, 8
Pellet count	62 ± 5	76 ± 4	74 ± 7	88 ± 7	0.977	0.0009	1, 8
Movement (cm/s)	3.2 ± 0.5	2.4 ± 0.7	3.1 ± 0.6	2.2 ± 0.7	0.703	0.1540	1, 10

Supplementary Table 1. *Daily bodyweight, food intake, water intake and faecal output values for control and LS lesioned mice.* Data corresponds to graphs in **Figure 5a**-**f**. All comparisons showed non-significant fixed effect interactions between treatment and time. Thus, LS lesions did not significantly affect any of the measurements presented.

	Control		TL lesion		P (time x treatment)	F	DFn, DFd
	Baseline	Post surg.	Baseline	Post Surg.			
Bodyweight (g)	21.2 ± 0.2	21.2 ± 0.7	21.3 ± 1.4	21.5 ± 1.5	0.434	0.6779	1, 8
Food intake(g)	3.8 ± 0.4	4.0 ± 0.4	4.0 ± 0.8	4.2 ± 0.3	0.803	0.0665	1, 8
Water intake (ml)	4.3 ± 0.4	4.4 ± 0.2	4.1 ± 1.0	4.6 ± 0.6	0.258	1.482	1, 8
Faeces (dry g)	0.7 ± 0.08	0.7 ± 0.09	0.6 ± 0.07	0.7 ± 0.11	0.009	11.97	1, 8
Pellet count	74 ± 6	79 ± 9	70 ± 10	74 ± 12	0.878	0.0253	1, 8
Movement (cm/s)	2.8 ± 0.7	2.7 ± 0.7	3.4 ± 0.4	3.4 ± 0.7	0.911	0.0135	1, 7

Supplementary Table 2. *Daily bodyweight, food intake, water intake and faecal output values for control and TL lesioned mice.* Data corresponds to graphs in **Figure 5g**-**l**. All but a single comparison showed non-significant fixed effect interactions between treatment and time. Daily average faecal mass output showed a significant time x treatment interaction (P = 0.009, two-way, repeated measures ANOVA, N = 5 animals in each group). Despite this, there was no significant differences between the TL lesion and control groups in daily faecal output mass by post hoc analysis at either the baseline or the post-surgery time points (P = 0.147 and 0.980, respectively, Sidak post-tests). Daily pellet counts were also not significantly different between control and TL lesion groups. Taken together, these data indicate no significant differences between the control and TL lesioned mice for any of the in vivo measurements.

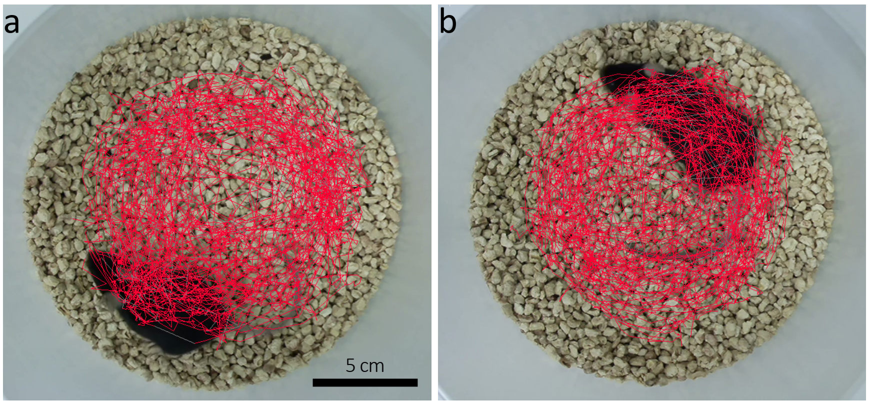

Supplementary Figure 1. *Ambulation before and after DRG surgical removal.* **a – b)** Representative tracking of control and LS lesioned mice over 30 minutes at 6 months post-surgery, respectively.

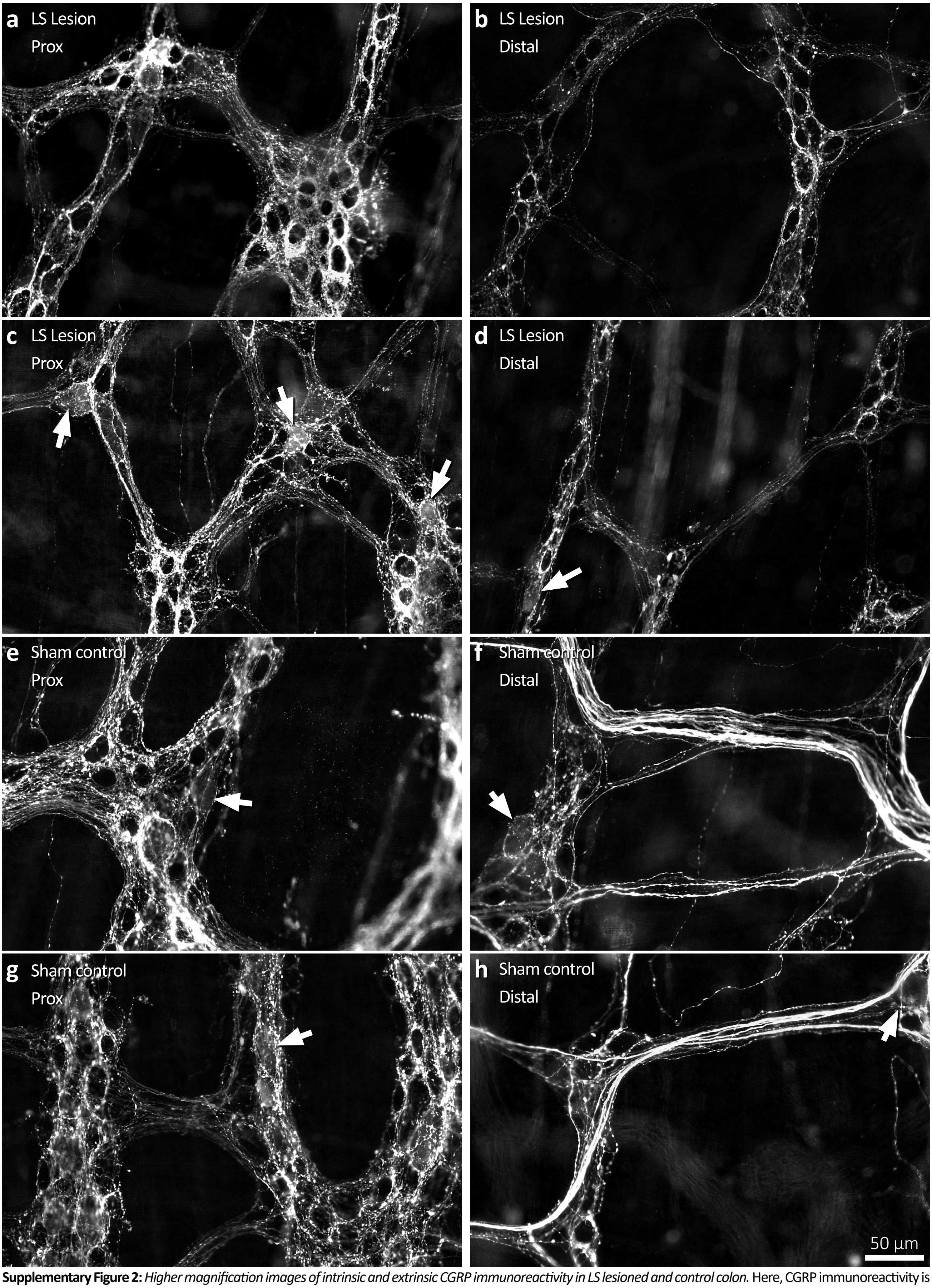

Supplementary Figure 2: *Higher magnification images of intrinsic and extrinsic CGRP immunoreactivity in LS lesioned and control colon.* Here, CGRP immunoreactivity is shown in micrographs from LS lesioned (**a - d**), and sham control (**e - h**) proximal and distal colons. In proximal colon, there was little difference in CGRP immunoreactivity between lesioned and control animals. In both cases, CGRP was abundant in varicose nerve fibres that often appeared to densely surround the locations of unlabelled enteric nerve cell bodies. Occasional nerve cell bodies were also faintly labelled with CGRP antisera (arrows). In distal colon, the major difference between LS lesioned animals compared to control was the absence of large smooth, intensely immunoreactive axons that are characteristic of extrinsic nerve fibres. This type of axon is clearly visible in **f**) and **h**). Faintly immunoreactive nerve cell bodies were present in both LS lesioned and control distal colon (arrows). The intrinsic CGRP immunoreactivity that persisted in LS lesioned distal colon (**b** and **d**) comprised predominantly of fine varicose nerve fibres.

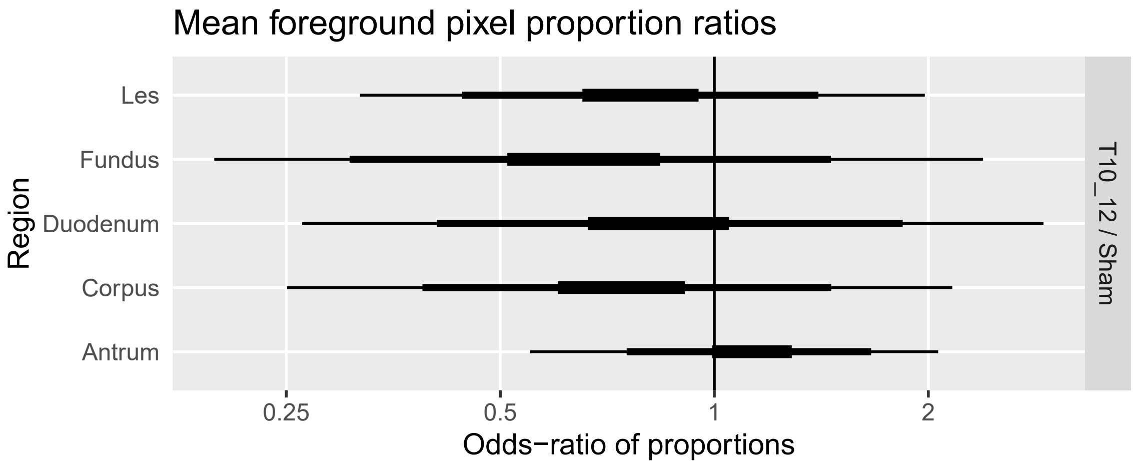

Supplementary Figure 3. *Quantification of relative changes in CGRP immunoreactivity in the stomach, small bowel and lower esophageal sphincter, following surgical removal of T10-12 DRG.* The horizontal lines from thickest to thinnest depict the 50%, 95%, and 99.5% posterior intervals of the change in CGRP density of lesion vs sham. This graph shows that the no-effect ratio of 1 is well within all 95% intervals, indicating that the lesion had no detectable effect on the CGRP density in all regions of the stomach, duodenum and esophagus.

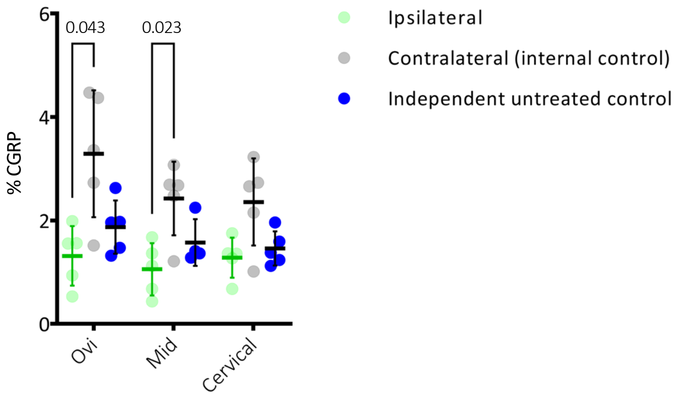

Supplementary Figure 4: *CGRP density after unilateral T13-L2 (TL) lesions: comparison with both an internal control (contralateral uterine horn) and an independent non-surgical control group.* The addition of an independent non-surgical control group reduced statistical significance of all pairwise comparisons. Statistically significant differences remained at the oviduct end and mid region of the uterus, but not the cervical end (two-way repeated measures ANOVA, Tukey post-tests, n = 5 TL lesioned animals and 5 non-surgical animals). Interestingly, the independent control group showed no significant difference to either the ipsilateral or contralateral uterine horns in the lesioned animals, with values that were intermediate to both groups. This raises the possibility that TL DRG lesions may not only cause loss of CGRP-containing afferents in the target organ, but also induce compensatory changes in remaining spinal afferents that lead to an increase in CGRP immunoreactivity. Error bars represent mean ± SD, individual markers represent individual animal averages.
