# Supplementary material for: Disengaging spinal afferent nerve communication with the brain in live mice
Source: Commun Biol. 2022 Sep 14;5:915. doi: 10.1038/s42003-022-03876-x (PMC9475039; doi:10.1038/s42003-022-03876-x)
Supplement: Supplementary file 3 — Reporting Summary [file 42003_2022_3876_MOESM3_ESM.pdf]

Corresponding author(s): DBPR COMMSBIO-22-0159D

Last updated by author(s): Aug 8, 2022

Reporting Summary

Nature Portfolio wishes to improve the reproducibility of the work that we publish. This form provides structure for consistency and transparency in reporting. For further information on Nature Portfolio policies, see our Editorial Policies and the Editorial Policy Checklist.

Statistics

For all statistical analyses, confirm that the following items are present in the figure legend, table legend, main text, or Methods section.

n/a	Confirmed	
☐	☒	The exact sample size (n) for each experimental group/condition, given as a discrete number and unit of measurement
☐	☒	A statement on whether measurements were taken from distinct samples or whether the same sample was measured repeatedly
☐	☒	The statistical test(s) used AND whether they are one- or two-sided<br>*Only common tests should be described solely by name; describe more complex techniques in the Methods section.*
☒	☐	A description of all covariates tested
☐	☒	A description of any assumptions or corrections, such as tests of normality and adjustment for multiple comparisons
☐	☒	A full description of the statistical parameters including central tendency (e.g. means) or other basic estimates (e.g. regression coefficient) AND variation (e.g. standard deviation) or associated estimates of uncertainty (e.g. confidence intervals)
☐	☒	For null hypothesis testing, the test statistic (e.g. F, t, r) with confidence intervals, effect sizes, degrees of freedom and P value noted<br>*Give P values as exact values whenever suitable.*
☐	☒	For Bayesian analysis, information on the choice of priors and Markov chain Monte Carlo settings
☒	☐	For hierarchical and complex designs, identification of the appropriate level for tests and full reporting of outcomes
☒	☐	Estimates of effect sizes (e.g. Cohen's d, Pearson's r), indicating how they were calculated

Our web collection on statistics for biologists contains articles on many of the points above.

Software and code

Policy information about availability of computer code

Data collection	LabChart (v 7; ADInstruments)<br>Spike2 (v 7.20; Cambridge Electronic Design)<br>AnalySIS Image (v 5.0; Olympus-SIS,Münster, Germany)<br>NDPview2 (v U12388-21)
Data analysis	Fiji 354 Image J (v 1.52p)<br>LabChart (v 7; ADInstruments)<br>Spike2 (v 7.20; Cambridge Electronic Design)<br>PyCharm (v 2019.3.4)<br>QuPath (v 0.1.2)<br>GraphPad Prism (v 9.3.1)

For manuscripts utilizing custom algorithms or software that are central to the research but not yet described in published literature, software must be made available to editors and reviewers. We strongly encourage code deposition in a community repository (e.g. GitHub). See the Nature Portfolio guidelines for submitting code & software for further information.

Data

Policy information about availability of data

All manuscripts must include a data availability statement. This statement should provide the following information, where applicable:

- Accession codes, unique identifiers, or web links for publicly available datasets
- A description of any restrictions on data availability
- For clinical datasets or third party data, please ensure that the statement adheres to our policy

The datasets generated during and/or analysed during the current study are available from the corresponding author on reasonable request.

Field-specific reporting

Please select the one below that is the best fit for your research. If you are not sure, read the appropriate sections before making your selection.

☒ Life sciences ☐ Behavioural & social sciences ☐ Ecological, evolutionary & environmental sciences

For a reference copy of the document with all sections, see nature.com/documents/nr-reporting-summary-flat.pdf

Life sciences study design

All studies must disclose on these points even when the disclosure is negative.

Sample size	Sample sizes were based on 25% of measurement variance explained by the treatment effect (the minimum effect of interest), and a within group variance of 20% (effect size f = 1.12). Repeated measures ANOVA with 2 groups with an alpha error probability of 5% and power of 95% gives a minimum sample size of 5 replicates in each group.
Data exclusions	No data were excluded.
Replication	Standard replication of measurements were performed for all experiments. Depletion of colonic CGRP was additionally replicated at multiple time points with independent experimental cohorts.
Randomization	Mice were allocated to experimental or sham control groups in an alternating manner. The only selection criteria was based on age range (3-6 months); applied equally to both groups.
Blinding	Sham and experimental mice were externally indistinguishable, enabling blinding during data collection and analysis. Surgery and data collection/analysis were performed by independent researchers.

Reporting for specific materials, systems and methods

We require information from authors about some types of materials, experimental systems and methods used in many studies. Here, indicate whether each material, system or method listed is relevant to your study. If you are not sure if a list item applies to your research, read the appropriate section before selecting a response.

Materials & experimental systems

n/a	Involved in the study	
☐	☒	Antibodies
☒	☐	Eukaryotic cell lines
☒	☐	Palaeontology and archaeology
☐	☒	Animals and other organisms
☒	☐	Human research participants
☒	☐	Clinical data
☒	☐	Dual use research of concern

Methods

n/a	Involved in the study	
☒	☐	ChIP-seq
☒	☐	Flow cytometry
☒	☐	MRI-based neuroimaging

Antibodies

Antibodies used	Anti-CGRP (polyclonal, raised in rabbit against the full length rat alpha CGRP peptide; Bachem, cat #T-4032, formerly "IHC-6006" from Peninsula Laboratories International Inc.; lot A17601).<br>Donkey anti-rabbit Cy3; 7.5 µg/ml; Jackson ImmunoResearch Laboratories Inc. cat #711-165-152; lot 133645
Validation	The CGRP antibody recognized a single band of ~14 kDa, corresponding to CGRP's molecular weight, on western blots of horse ileum homogenates (Russo et al., 2010). Preabsorption of the diluted antibody with the immunogen abolishes staining in horse spinal cord, dorsal root ganglia, and intestine (Domeneghini et al., 2004).

Domeneghini C, Radaelli G, Arrighi S, Bosi G, Dolera M (2004) Cholinergic, nitrergic and peptidergic (Substance P-and CGRP-utilizing) innervation of the horse intestine. A histochemical and immunohistochemical study. Histology and Histopathology 19:357-370.
Russo D, Bombardi C, Grandis A, Furness JB, Spadari A, Bernardini C, Chiocchetti R (2010) Sympathetic innervation of the ileocecal junction in horses. Journal of Comparative Neurology 518:4046-4066

Animals and other organisms

Policy information about studies involving animals; ARRIVE guidelines recommended for reporting animal research

Laboratory animals	Mice C57BL6/J of either sex; 3-6 months old
Wild animals	No wild animals.
Field-collected samples	No field-collected samples.
Ethics oversight	Procedures were approved by the Animal Welfare Committee of Flinders University (approvals #861-13 and #933-16).

Note that full information on the approval of the study protocol must also be provided in the manuscript.
